# Human inborn errors of the alternative NF-κB pathway

**DOI:** 10.70962/jhi.20250104

**Published:** 2025-11-21

**Authors:** Tom Le Voyer, Jean-Laurent Casanova, Anne Puel

**Affiliations:** 1 Laboratory of Human Genetics of Infectious Diseases, Necker Branch, INSERM UMR 1163, Paris, France; 2 Imagine Institute, Paris Cité University, Paris, France; 3 St. Giles Laboratory of Human Genetics of Infectious Diseases, Rockefeller Branch, Rockefeller University, New York, NY, USA; 4 Clinical Immunology Department, Assistance Publique Hôpitaux de Paris, Saint-Louis Hospital, Paris, France; 5 Pediatric Hematology-Immunology Unit, Necker Hospital for Sick Children, Paris, France; 6 Howard Hughes Medical Institute, New York, NY, USA

## Abstract

Inborn errors of the “core” components of the alternative NF-κB pathway—NIK, IKK-α, RelB, and NF-κB2—underlie various T and/or B cell deficiencies, frequently associated with syndromic features, including ectodermal dysplasia and lymph node hypoplasia. Their impact on medullary thymic stromal cells (mTECs) also underlies the development of autoantibodies neutralizing type I interferons (IFNs), conferring a predisposition to severe viral diseases. Inborn errors of “upstream” ligands or surface receptors engaging this pathway affect secondary lymphoid organ organization (LTβR), B cell development and survival (BAFFR), T cell and antigen-presenting cell costimulation (CD40L/CD40), or osteoclast differentiation (RANK/RANKL). Finally, inborn errors of TRAF3, a negative “regulator” of this pathway, underlie immune dysregulation, infection, and lymphoproliferation. Various inborn errors of the human alternative NF-κB pathway have, thus, delineated the essential and redundant roles of its components in leukocytic and non-leukocytic cells.

## Teaser

This review synthesizes human inborn errors of the alternative NF-κB pathway-affecting core components, upstream ligands/receptors, or regulators, or regulatory elements-highlighting their shared and distinct effects on lymphoid development, immune dysregulation, and mTEC-mediated tolerance, which can lead to impaired B and T cell function, the development of neutralizing anti-type I IFN autoantibodies, susceptibility to severe viral diseases, and syndromic features.

## Graphical Abstract

**Figure:**
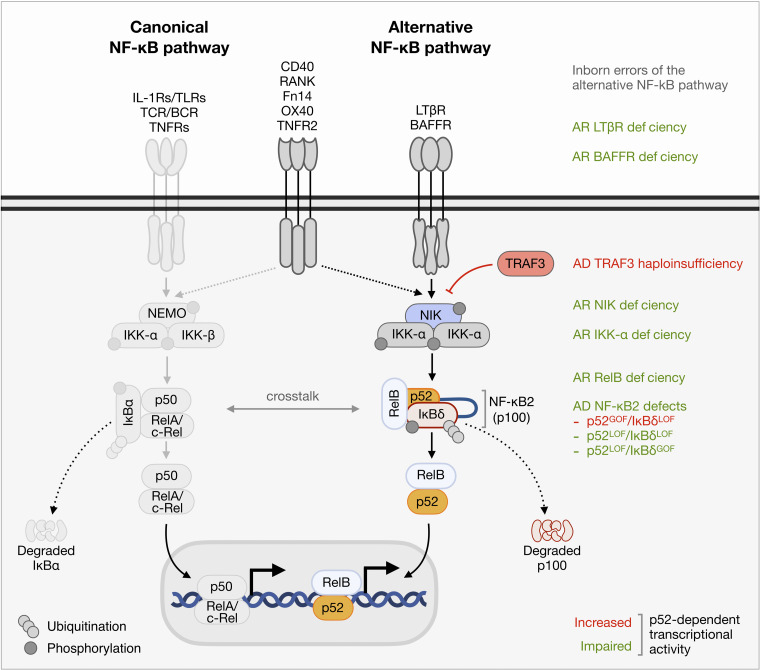

## The alternative NF-kB pathway in mouse and human cells

The human and mouse NF-κB family comprises five transcription factors: NF-κB1 (p105/p50), NF-κB2 (p100/p52), RelA (p65), RelB, and c-Rel (1). These structurally related proteins exert their biological activities by forming homo- or heterodimers. All five members of the family are strongly expressed in blood and lymphoid tissues, such as the spleen and lymph nodes, and their levels are highest in T and B cells. NF-κB2 and RelB are also strongly expressed by medullary thymic epithelial cells (mTECs). The canonical (or classical) NF-κB pathway can be activated by various receptors, including the T and B cell receptors (TCR and BCR), and members of the interleukin-1 receptor/toll-like receptor (TLR) and tumor necrosis factor receptor (TNFR) superfamilies. Upon activation, the inhibitor of nuclear factor kappa-B (IκB) kinase (IKK) complex, comprising IKK-α, IKK-β, and NEMO/IKK-γ (2), phosphorylates inhibitors, such as IκB-α and p105. This phosphorylation promotes their ubiquitination and targeting for proteasomal degradation, thereby releasing free cytoplasmic p50/RelA or p50/c-Rel heterodimers for translocation to the nucleus, where they regulate the transcription of genes involved in immune responses, cell development, and survival. This pathway is tightly regulated, resulting in rapid but transient responses (2, 3).

By contrast, the alternative (or noncanonical) NF-κB pathway operates with slower but sustained activation (4). It is triggered by a narrow range of receptors from the TNFR superfamily (TNFRSF) expressed predominantly on stromal cells, such as TNFRSF12A (TWEAK receptor) and lymphotoxin β receptor (LTβR), or on leukocytes, including B cell-activating factor receptor (BAFFR), receptor activator of NF-κB (RANK), and CD40 (Table 1). Most of these receptors can also induce rapid and transient activation of the canonical NF-κB pathway, thereby mediating biological processes dependent on functional cooperation between the two NF-κB pathways. Mouse studies have revealed that BAFFR is crucial for B cell development beyond the transitional T2 stage, B cell survival, immunoglobulin (Ig) IgG and IgM production, and initiation of the germinal centers (GCs) reaction (5, 6). They have also shown that CD40−CD40 ligand (CD40L) engagement provides an essential costimulatory signal for B cell proliferation and Ig production *in vitro *(7, 8) and is required for GC organization, antibody isotype switching, and the response to T-dependent (TD) antigens *in vivo* (7). Finally, these studies have demonstrated that LTβR expression on stromal cells in secondary lymphoid organs (SLOs), including lymph nodes and Peyer’s patches, is crucial for correct structural organization (9), and that the expression of CD40 and RANK on mTECs is required for their maturation and the expression of AIRE (10), a key regulator of the negative selection of self-reactive thymocytes (central tolerance).

**Table 1.** TNFRSF and the corresponding ligands involved in the activation of the alternative NF-κB pathway across various human tissues and cell types

Ligand (*gene*)	Tissue expression	Cellular expression	Receptor (*gene*)	Tissue expression	Cellular expression	Reference
CD40L or CD154 (*CD40LG*)	SLOs and thymus	Leukocytes: **activated T cells** (CD4^+^, CD8^+^, and γδ), NKT, MAIT, Tregs, also detected on NK cells, mast cells, basophils, and eosinophils	CD40 or TNFRSF5 (*CD40*)	SLOs and thymus	Leukocytes: **B cells, dendritic cells**, monocytes, and macrophages. Non-hematopoietic cells: epithelial cells (mTECs), follicular dendritic cells, endothelial cells, and fibroblasts	(11, 12)
LTα1β2 (*LTA* and *LTB*)	SLOs, thymus, and bone marrow	Leukocytes: **activated B** and T cells, NKT, MAIT, Tregs, and dendritic cells	LTβR or TNFRSF3 (*LTBR*)	SLOs, thymus, and bone marrow	Leukocytes: dendritic cells, monocytes, and macrophages. Non-hematopoietic cells: **LN stromal cells** (FRCs, including MRCs^a^), epithelial cells, endothelial cells, and mesenchymal cells	(12, 13, 14)
LIGHT (*TNFSF14*)	Broad, including bone marrow, SLOs, and liver	Leukocytes: T cells, NK cells, macrophages, monocytes, and dendritic cells				
			LIGHT-R or HVEM (*TNFRSF14*)	Broad, including SLOs	B and T lymphocytes, NK cells, monocytes, macrophages, and dendritic cells	(12, 15)
RANKL (*TNFSF11*)	SLOs, bone marrow, bones, and thymus	Leukocytes: activated T and B cells and NK cells. Non-hematopoietic cells: **osteoblasts**, osteocytes, LN stromal cells (MRCs^a^), epithelial, and germ cells	RANK (*TNFRSF11A*)	Gastrointestinal tract, SLOs, and bones	Hematopoietic cells: **osteoclasts**, macrophages, dendritic cells, and NK cells. Non-hematopoietic cells: thymic epithelial cells (mTECs), intestinal epithelial cells (enterocytes, microfold cells), lymphatic and endothelial cells (LEC)	(12, 14, 16, 17)
BAFF or BLys (*TNFSF13B*)	SLOs and bone marrow	Leukocytes: monocytes, macrophages, dendritic cells, and neutrophils. Non-hematopoietic cells: **LN stromal cells** (FRCs, including TRCs^a^ and MRCs^a^)	BAFFR (*TNFRSF13C*)	SLOs and bone marrow	Leukocytes: **mature B cells** (transitional, naïve, GC, and memory B cells)	(12, 14, 18, 19)
TWEAK (*TNFSF12*)	Broad, including bone marrow, SLOs, and liver	Leukocytes: T and B^a^ cells, NK cells, monocytes, macrophages, dendritic cells, and neutrophils Non-hematopoietic cells: epithelial cells and endothelial cells	Fn14 or TWEAK-R (*TNFRSF12A*)	Broad, including SLOs	Non-hematopoietic cells: epithelial cells	(12, 20)

Bold typeface is used to indicate the principal cell type in which expression occurs. FRC, fibroblastic reticular cells; LEC, lymphatic endothelial cells; LN, lymph node; MRC, marginal reticular cells; TRC, T-zone reticular cells.

a Evidence only at the mRNA level.

**Figure 1.**
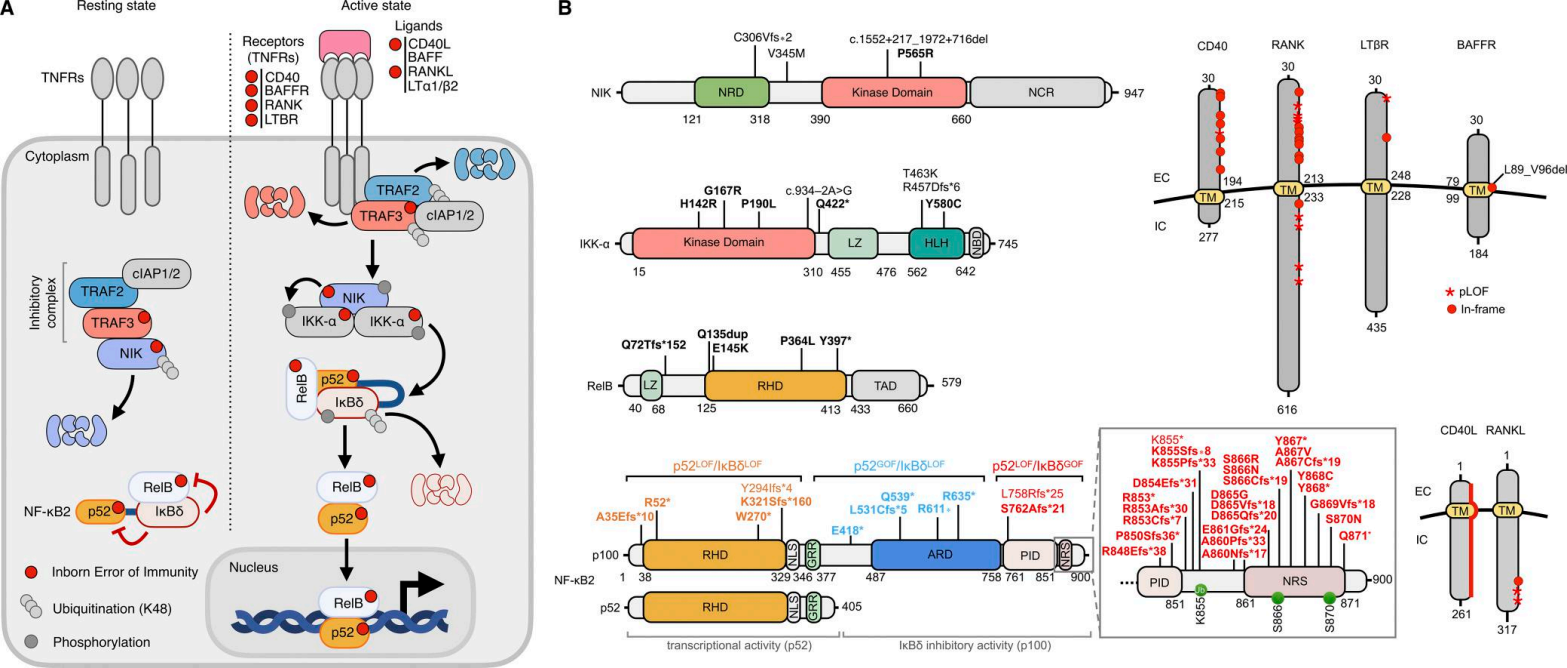
**Schematic representation of the alternative NF-κB pathway and its components affected by germline mutations.** **(A)** Components and regulation of the alternative NF-kB signaling pathway, with the corresponding human inborn errors, indicated by red dots. **(B)** Schematic representation of the proteins and their corresponding reported variants. ARD, ankyrin repeat domain; HLH, helix-loop-helix; KD, kinase domain; LZ, leucine zipper; NBD, NEMO-binding domain; NCR, non-catalytic region; NRD, negative regulatory domain; NRS, NIK-responsive sequence; PID, processing inhibitory domain; RHD, Rel homology domain; TAD, transactivation domain.

Upon ligand binding, activation of the human noncanonical NF-κB signaling pathway leads to the recruitment and sequestration of tumor necrosis factor receptor-associated factor 2 (TRAF2) and TRAF3 by the receptor (Fig. 1 A) (4). This stabilizes the NF-κB-inducing kinase (NIK) protein, which is otherwise constitutively degraded. The accumulated NIK phosphorylates and activates IKK-α, which in turn phosphorylates the precursor form of NF-κB2 (p100) at Ser866/870 and Ser872 (21). Phosphorylated p100 undergoes ubiquitination and proteasomal processing to generate the active p52 subunit, leading to preferential formation of the transcriptionally active p52/RelB heterodimer (4). This heterodimer translocates to the nucleus, where it binds to κB sites, thereby regulating the transcription of multiple target genes. In the basal state, unprocessed p100 inhibits transcription by forming a cytoplasmic complex with p52 and RelB (known as kappaBsomes), thereby preventing their translocation to the nucleus; this function is referred to as the IκBδ activity of p100 (22, 23). Inborn errors of NIK, IKK-α, RelB, NF-κB2, and TRAF3, along with defects of upstream receptors or their ligands, have been identified in humans (Fig. 1 A) (24, 25, 26, 27, 28, 29). Here, we review current knowledge from studies of human inborn errors of immunity (IEIs) of the alternative NF-κB pathway (30) and compare the phenotypic profiles of patients with those of the corresponding murine models to delineate more precisely the intrinsic contributions of the stromal and leucocytic compartments.

## Human inborn errors of core components of the alternative NF-κB pathway

### Autosomal recessive (AR) NIK deficiency

NIK was the first component of the noncanonical NF-κB pathway to be identified (21). This kinase is a key upstream regulator, controlling the phosphorylation of IKK-α and p100 to generate p52 (4). Biallelic loss-of-function (LOF) variants of *MAP3K14* have been reported in eight patients from Iran, Saudi Arabia, South Africa, and Turkey (24, 27, 31, 32) (Table 1). All variants except the p.V345M missense variant affect the kinase domain of NIK (AA 390–660) through truncation (p.C306Vfs∗2), deletion (c.1552+217_1972+716del), or missense (p.P565R) (24, 27, 31, 32) mechanisms (Fig. 1 B). All the patients had combined immunodeficiency (CID), characterized by recurrent bacterial sinopulmonary infections (*n* = 7), disseminated Bacillus Calmette–Guérin (BCG) (*n* = 3), viral (CMV, *n* = 2; norovirus, *n* = 2), fungal (chronic mucocutaneous candidiasis [CMC, *n* = 6]), and parasitic (*Cryptosporidium*, *n* = 2) diseases (24, 27, 31, 32, and Table 2). The patients lacked tonsils and displayed lymph node hypoplasia, as shown by lymphoscintigraphy (32). Most also had low levels of IgM (*n* = 7/8), IgG (*n* = 7/8), and IgA (*n* = 8/8), with poor responses to TD vaccines. This is consistent with mouse models, which lack SLOs and display impaired GC formation and defective TD immune responses due to a GC-intrinsic stromal defect (33, 34, 35, 36). Immune phenotyping of the patients showed that total T cell counts were normal, with normal (*n* = 2/6) or high (*n* = 4/6) proportions of naïve CD4^+^ T cells, normal T cell receptor excision circle (TREC) levels, and normal levels of T cell proliferation in response to mitogens or antigens *in vitro* (24, 32). The proportion of regulatory T cells (Tregs) was normal, whereas that of T follicular helper (Tfh) cells was low (24). Natural killers (NK) cell proportions were low (*n* = 3/8) to normal (*n* = 5/8). B cell counts ranged from low (*n* = 4/8) to normal (*n* = 4/8), with an abnormally small proportion of CD27^+^ memory B cells (*n* = 6/6) (24, 32). An analysis of the rearranged *IGHV* of IgG and IgA isotypes revealed lower rates of somatic hypermutation (SHM) than in HC, although as the decrease in SHM rates was smaller than that in CD40L-deficient patients (24). Such deficiencies are treated by IgG supplementation and antibiotic prophylaxis. Four patients underwent hematopoietic stem cell transplantation (HSCT), with two survivors achieving full cellular and humoral reconstitution (24, 27, 32).

**Table 2.** Shared and distinct clinical features of patients with inborn errors of the alternative NF-κB pathway

​		AD NF-κB2 haploinsufficiency (p52^LOF^/IκBδ^LOF^)	AD p52-GOF (p52^GOF^/IκBδ^LOF^)	AD IκBδ-GOF (p52^LOF^/IκBδ^GOF^)	AR NIK deficiency	AR IKK-α deficiency	AR RelB deficiency	AD TRAF3 haploinsufficiency	AR BAFFR deficiency	AR LTBR deficiency	XL-CD40L or AR CD40 deficiency	AR RANK or RANKL deficiency
Gene		*NFKB2*	*NFKB2*	*NFKB2*	*NIK*	*CHUK*	*RELB*	*TRAF3*	*BAFFR*	*LTBR*	*CD40/CD40L*	*RANK/RANKL*
IEI/disease group		PAD	PAD	PAD	CID	CID	CID	Immune dysregulation	PAD	CID	CID	Osteopetrosis
Immunological phenotypes	B cells	↓ Ig, ↓ B cells, and ↑ mem B cells	↓ Ig and ↓ B cells	↓↓↓ Ig and ↓↓↓ B cells	↓↓↓ Ig and ↓↓↓ B cells	↓↓ Ig and ↓↓ B cells	↓↓ Ig and ↓↓ B cells	↑↑ Ig, ↑↑ B cells, and ↓↓ mem B cells	↓↓↓ Ig and ↓↓↓ B cells	↓↓↓ Ig and ↓↓↓ mem B cells	↑↑ IgM, ↓↓↓ IgG, and ↓↓↓ mem B cells	↓ Ig and ↓ mem B cells
	T cells	Normal	Normal	↑ CD4^+^ naïve T, ↓ cTFh, and ↓ Treg	↑/↓ CD4^+^ naïve T and ↓ cTFh	↑ CD4^+^ naïve T, ↓ cTFh, and ↓ Treg	↓ CD4^+^ naïve T	↓ CD4^+^ naïve T, ↑ cTFh, and ↑ Treg	Normal	↓ cTFh and ↓ Treg	↓ cTFh	Normal
Susceptibility to infection	Respiratory tract bacterial infections	++	++	+++	+++	++	++	+++	++	+++	+++	±
	SARS-CoV-2, influenza, VZV, HSV-1	-	-	+++	++	+++	++	-	-	-	-	-
	Candidiasis (CMC)	-	-	-	+++	++	++	-	-	-	-	-
	Mycobacterial	-	-	-	++	-	-	-	-	-	-	-
	Cryptococcal	-	-	-	++	-	+	-	-	-	++	-
Syndromic features	Pituitary defects	-	-	++	-	-	-	-	-	-	-	-
	Alopecia areata/totalis	-	-	++	-	++	-	-	-	-	-	-
	Nail dystrophy	-	-	++	-	++	-	-	-	-	-	-
	Ectodermal dysplasia (sparse hair, eyebrows, or eyelashes)	-	-	+	-	++	-	-	-	-	-	-
SLO development	Lymph nodes	Normal	Lymphadenopathy	Normal	Hypoplasia	Hypoplasia	Hypoplasia	Lymphadenopathy	Normal	Hypoplasia	Normal/hypoplasia	Hypoplasia
	Spleen	Normal	Normal	Normal	Normal	Normal	Normal	Splenomegaly	Normal	Functional asplenia	Normal	Normal
Autoimmunity	Organ-mediated autoimmunity	++	+	++	-	++	++	++	-	-	-	-
	Auto-Abs against type I IFNs	Negative	Negative	Positive	Positive	Positive	Positive	Negative	Negative	Negative	Negative	Negative

+, occasional; ++, commonly observed; +++, very frequent; CID, combined immunodeficiency; CMC, chronic mucocutaneous candidiasis; cTFh, circulating T follicular helper; mem, memory; PAD, predominantly antibody deficiency; SLOs, secondary lymphoid organs.

Functional testing in overexpression systems showed that the p.P565R variant lacks kinase activity, whereas the p.V345M variant appears to be hypomorphic (24, 31). The other alleles (p.C306Vfs∗2 and c.1552+217_1972+716del) have not undergone functional testing. Patient-derived Epstein–Barr virus (EBV)-immortalized B lymphoblastoid cell lines displayed an almost total abolition of the processing of p100 to generate p52, with almost no p52 detected in the nucleus following BAFF stimulation (24). By contrast, the response of patient B cells to CD40L and IL-4 was only slightly weaker than that of healthy individuals, in terms of the upregulation of costimulatory molecules, class-switch recombination (CSR), and proliferation. However, B cell proliferation in response to CD40L combined with IL-21, and ICOSL upregulation upon CD40L stimulation, were markedly reduced (24). In addition to playing a role in B cell terminal differentiation and antibody affinity maturation, CD40 signaling in myeloid cells is essential for antimycobacterial immunity through IL-12 secretion (37, 38). This may explain the susceptibility to weakly virulent mycobacterial diseases observed in patients with AR NIK deficiency. In addition to B, T, and possibly myeloid cells, AR NIK deficiency also affects alternative NF-κB pathway activation in stromal cells, as shown in mice (39, 40, 41). Consistently, patient fibroblasts display a severe impairment of p100 phosphorylation and processing into p52, p52 nuclear translocation, and the upregulation of *VCAM1* and *CCL20* transcripts in response to LTα1β2 (Lt) stimulation (24, 32). The early activation of the canonical NF-κB pathway downstream from LTβR was also partly impaired, as shown by the low levels of IκBα degradation and p50 translocation to the nucleus upon Lt stimulation (24).

Consistent with the essential role of NIK in mTEC maturation and AIRE expression demonstrated in *Nik*-deficient mice (39, 40, 42, 43) (Table S2), patients with AR NIK deficiency have an abnormal thymic architecture, with few AIRE-expressing mTEC. AIRE is critical for T cell–mediated central tolerance to type I IFNs in humans (44). Consequently, these patients develop autoantibodies neutralizing type I IFNs (AAN-I-IFNs) (27). The persistence of AAN-I-IFNs years after transplantation suggests that their development results from NIK deficiency in mTECs (27). However, unlike the broader autoimmune manifestations observed in *Nik*-deficient mice, human NIK deficiency is associated with a relatively narrow spectrum of autoantigens, predominantly targeting type I IFNs, without features of organ-specific autoimmune diseases. Despite the small number of reported cases, studies of patients with AR NIK deficiency suggest that human NIK is essential for the activation of p52/RelB heterodimers in B and stromal cells. Mycobacterial diseases are reported in patients with inborn errors of the canonical NF-κB pathway affecting the IL-12/IFN-γ circuit, including X-linked (XL) NEMO, AR IKK-β, and AR c-Rel deficiencies [37, 38, 45], and opportunistic intestinal parasitic infections (cryptosporidiosis and microsporidiosis) are seen in patients with inborn errors of IL-21 signaling (AR IL-21R deficiency) or the CD40-dependent dendritic cell–T cell synapse (AR CD40, XL-CD40L, AR MHC class II deficiency, AR c-Rel deficiency, and AR FLT3L deficiency) (38, 46, 47, 48). Interestingly, such diseases have not been reported in patients with AR RelB deficiency or IKK-α deficiency, suggesting that NIK plays a broader role in host immunity, potentially involving the canonical NF-κB pathway—either by directly regulating canonical NF-κB dimers or through the accumulation of unprocessed p100 (49, 50, 51).

### AR IKKα deficiency

Human biallelic null variants of *CHUK*, encoding IKK-α, cause Cocoon syndrome, an embryo-lethal encasement syndrome associated with severe developmental malformations, including abnormally large cranial cysts, abnormal brain structure, and hypoplastic limbs (25). This condition has been reported in two fetuses homozygous for a nonsense (p.Q422*) *CHUK* variant, resulting in a complete lack of IKK-α protein (25) (Fig. 1 B). This phenotype closely resembles that of *Ikka* knockout (KO) mice, which have multiple severe skeletal and epidermal defects and die shortly after birth (52, 53), a phenotype not observed in *Nik*-KO mice (Table S2). These observations highlight the essential, NIK-independent role of human IKK-α in keratinocyte differentiation and epithelial development during embryogenesis. In addition, seven patients from five unrelated kindreds homozygous (*n* = 3, p.G167R [54], p.Y580C [55], and c.934–2A>G [56]) or compound heterozygous (*n* = 2, p.H142R/p.P190L [57, 58] and p.R457Dfs*6/p.T463K [59]) for *CHUK* variants have been reported (Fig. 1 B). These patients originated from Belgium, Canada, Italy, Saudi Arabia, and Turkey and presented with ectodermal dysplasia and/or immunodeficiency (54, 57, 58, 59). Three patients had syndromic clefting, including skeletal malformations and classic features of ectodermal dysplasia manifesting at birth, such as alopecia totalis or areata, absent or sparse eyebrows and eyelashes, hypohidrosis, or abnormal dental enamel (56, 57, 59). One patient homozygous for the essential splice site c.934–2A>G variant was diagnosed with Bartsocas–Papas syndrome (56), a milder form of Cocoon syndrome, and two patients compound heterozygous for the R457Dfs*6/T463K or H142R/P190L variants were diagnosed with Hay–Wells/ankyloblepharon-ectodermal dysplasia-clefting syndrome (57, 59). All six patients evaluated developed CID within their second year of life, with hypogammaglobulinemia and recurrent bacterial diseases (e.g., otitis and respiratory tract) (56, 57, 59) (Table S1). They were also susceptible to viral diseases (rotavirus, coxsackievirus, respiratory syncytial virus [RSV], SARS-CoV-2, adenovirus, bocavirus, rhinovirus, CMV, and epidermodysplasia verruciformis; *n* = 7) (54, 55, 57, 59), CMC (*n* = 3) (54, 55), and *Salmonella enterica* infections (*n* = 2) (54, 55). Additional features included an absence of tonsils or palpable lymph nodes (*n* = 5/5), as shown by lymphoscintigraphy (54, 55, 57), chronic hepatitis with periportal lymphocytic infiltration, and fibrosis (*n* = 5) (54, 55, 57), leading to liver transplantation (*n* = 2) (54, 55), autoimmune enteropathy (*n* = 2) (54, 55), and diffuse large B cell lymphoma (DLBCL) (*n* = 1) (57). These phenotypes resemble those observed in *Ikka*-deficient mice and mTEC-specific *Ikka*-KO mice, which display disorganized SLOs and T cell infiltration into organs (39, 52, 53). Three patients died (two after transplantation and one after chemotherapy for DLBCL) (54, 55, 57). HSCT was not performed. Most of the patients evaluated (*n* = 5/5) had hypogammaglobulinemia, with progressive B cell lymphopenia, low proportions of IgM^+^CD27^+^ marginal zone (MZ) and memory B cells, and high proportions of transitional B cells (54, 55, 57). The patients’ IgM repertoire displayed restricted diversity, skewed gene usage, and a low rate of SHM in the rearranged *IGHV* regions of the IgM heavy chain (54). The proportions of memory CD4^+^ and CD8^+^ T cells were low, with low levels of clonotype diversity and altered TCR variable gene usage (54, 55, 57). Blood counts of Tregs and Tfh were low to normal (54, 57), a pattern also observed in mTEC-specific *Ikka*- and *Nik*-KO mice (39). Finally, AAN-I-IFNs were detected in all patients evaluated (*n* = 4 from 2 kindreds) (54, 57).

Functional studies based on overexpression showed that the p.H142R, p.P190L, and p.G167R IKK-α variants of the kinase domain were expressed but LOF in terms of kinase activity (including autophosphorylation) (54, 57). These variants were also impaired in their interactions with NIK or p100 (54, 57) and in IKK-α–mediated p100 phosphorylation and processing (54, 57). By contrast, the p.Y580C variant, located in the helix-loop-helix domain, retained normal kinase activity and binding to p100, but lost its ability to bind NIK (55, 57). Patient fibroblasts displayed severely impaired processing of p100 into p52 (p.H142R/p.P190L, p.G167R, and p.Y580C) and defective RelB translocation to the nucleus and DNA binding (p.H142R/p.P190L and p.G167R), together with low levels of *VCAM1* upregulation upon Lt stimulation (p.Y580C), as observed in fibroblasts from patients with AR NIK deficiency (32). Interestingly, the translocation of p50-, RelA-, and RelB-dependent heterodimers to the nucleus upon TNF stimulation was partly impaired in patient SV40 fibroblasts, despite the normal degradation and phosphorylation of IκBα (57). This suggests that IKK-α can regulate the canonical NF-κB pathway through p50- or RelA-dependent dimer activation, as previously described for NIK (55). Patient IKK-α-deficient B cells displayed impaired proliferation, class switching to IgG, IgA, or IgE, and weaker ICOSL upregulation upon CD40 plus IL-4 stimulation (54), reminiscent of AR NIK deficiency (24). Together, these observations suggest that human IKK-α is essential for the NIK-dependent activation of p52/RelB dimers in stromal and B cells. It may also contribute to activation of the canonical NF-κB pathway through p50/RelA, at least in stromal cells, in addition to its role in keratinocyte and bone development.

### AR RelB deficiency

AR complete RelB deficiency was the first IEI reported to abolish the function of a REL/NF-κB family member. Nine patients from five kindreds originating from Canada (Irish descent), Israel (Iranian Jewish descent), Turkey, and China with biallelic rare or private variants of *RELB* (26, 60, 61, 62, 63) have been reported (Table S1). They are homozygous (*n* = 8 patients, 4 kindreds, p.Q72Tfs*152, p.Y397*, p.P364L, and p.Q135dup) or compound heterozygous (*n* = 1 patient, p.E145K/p.P364L) for *RELB* variants (Fig. 1 B). The in-frame variants are located in highly conserved regions of RelB, within the DNA-binding (p.E145K and p.Q135dup) or dimerization (p.P364L) domains of RelB. Like NIK-deficient patients, these patients had CID and suffered various infectious diseases within their first 3 years of life. They suffered from bacterial (recurrent otitis media, upper and lower respiratory tract infections, and *Salmonella* spp.), viral (HSV-1, varicella-zoster virus [VZV], adenovirus, epidermodysplasia verruciformis, and JC polyomavirus), and/or fungal (CMC, *Talaromyces marneffei*, and *Cryptococcus neoformans*) diseases. Two siblings homozygous for the P364L variant displayed T cell infiltration into organs, with dermatitis, hepatitis, gastritis, enteritis, and/or sclerosing cholangitis (62). One patient developed DLBCL (26). AR RelB deficiency results in an abnormal thymic architecture, characterized by a poorly formed medulla, a lack of Hassall’s corpuscles, and an absence of *AIRE*-expressing mTECs, similar to the effects observed in *Relb*-KO mice (27, 64) (Table S2). Consistently, seven of the eight patients tested had AAN-I-IFNs, consistent with their viral diseases, and these autoantibodies remained detectable 11 years after HSCT (26, 27). RelB-deficient patients had low to normal T cell counts, with impaired *in vitro* responses to activation with anti-CD3 antibodies or PHA, small numbers and proportions of naïve T cells, low proportions of recent thymic emigrants (RTEs), and an abnormally high proportion of Tregs (26, 62, 64). Unlike *Relb*-KO mice, which have nearly normal mature B cell counts (65), the patients displayed a progressive decline of B cell counts over time and low proportions of memory B cells (61, 62, 64). IgG and IgA levels were low, whereas three patients with AR complete RelB deficiency displayed transient increases in IgM levels (26, 64). All patients had impaired responses to conjugated vaccines (26, 64). Unlike patients with AR IKK-α and NIK deficiencies, these patients had normal or high proportions of Tregs and Tfh cells (26, 64). In addition, RelB-deficient patients had abnormally low proportions of mucosal-associated invariant T cells (MAIT) cells and normal proportions of γδ T cells (26).

Functional investigation of the patients’ alleles in an overexpression system showed that the p.Q72Tfs*152 and p.Y397* alleles were LOF in terms of p52/RelB-mediated transcriptional activity, whereas the remaining alleles were severely hypomorphic (26). Consequently, three patients from two kindreds were considered to have AR complete RelB deficiency, whereas another five patients from three kindreds had AR partial RelB deficiency. In patients’ fibroblasts, RelB expression was abolished (p.Q72Tfs*152 or p.Y397*), reduced (p.P364L), or normal (p.E145K/p.P364L) (26, 64), and patient leukocytes (p.Q135dup) displayed lower levels of RelB expression than control cells (61). All the patient fibroblasts tested displayed a severe defect of alternative NF-κB pathway activation, with little or no p52/relB heterodimer in the nucleus, together with weak *NFKB2* and *VCAM1* transcript upregulation following Lt stimulation (26). By contrast to what was observed in NIK-deficient fibroblasts, p100 phosphorylation and processing into p52 were preserved, as indicated by a normal p100/p52 ratio (26). In addition, upon Lt stimulation, levels of unphosphorylated p100 were markedly lower in RelB-deficient fibroblasts than in controls, consistent with the role of RelB in regulating *NFKB2* transcription and stabilizing p100 (23). By contrast to NIK- and IKK-α–deficient fibroblasts, RelB-deficient fibroblasts displayed no impairment of the translocation of RelA and p50 to the nucleus upon TNF stimulation, suggesting that RelB is not required for TNF-mediated activation of the canonical NF-κB pathway in human fibroblasts. The patients’ T cells proliferated poorly in response to mitogens or antigens (64). Consistent with observations for other IEIs with T cell–intrinsic defects of IL-17 immunity, RelB-deficient patients had abnormally small proportions of Th17 memory T cells and impaired IL-17A and IL-22 production, possibly accounting for their CMC (26). *In vitro*, B cell activation with CD40 plus IL-21 triggered extremely low levels of Ig secretion, despite normal proliferation upon CD40 engagement (26). These findings suggest that human RelB is essential for p52/RelB-dependent activation in B cells and stromal cells and that it may also be critical for IL-17–dependent mucocutaneous defense against *Candida*.

### AD inborn errors of NF-κB2

Human AD NF-κB2 disorders were first reported in 2013 (66), and over 130 cases in more than 27 countries have since been described (27, 67, 68, 69). To date, 38 deleterious variants of *NFKB2* have been identified in the heterozygous state (27, 67, 68, 69). These variants can be classified into three categories based on their localization and specific effects on p100 and p52 function, as determined by overexpression studies, corresponding to the three allelic forms of AD inborn errors of NF-κB2 (Fig. 1 B and Table S3). The first form accounts for most reported patients (*n* > 100), who are heterozygous for missense or nonsense variants located in the C-terminal region of p100, specifically affecting the NIK-responsive sequence (67). This region is crucial for the phosphorylation of p100 at serine residues 866 and 870 and its processing to generate p52 (21). To date, 28 C-terminal variants (including 6 missense and 22 truncating variants) impairing the processing of p100 to p52 have been reported (Fig. 1 B). These variants were inherited across two or more generations in two thirds of cases and occurred *de novo* in the remaining third. Approximately one third of patients carry the recurrent p.R853* variant, probably arising from a mutational hotspot rather than a founder effect, as this variant has been shown to occur *de novo* in several patients of diverse origins (27, 66, 69, 70, 71, 72, 73). These variants typically result in predominantly antibody deficiency (PAD) with complete penetrance before the age of 10 years (27, 67, 68) (Table S1). In addition, patients with C-terminal processing-resistant variants often present with distinctive features, such as hypopituitarism (observed in 38%) and ectodermal dysplasia, such as alopecia areata or totalis (30%), classically accompanied by additional signs of sparse hair, eyebrows, or eyelashes, and nail dysplasia (trachyonychia, 25%). Features of hypopituitarism and ectodermal dysplasia usually appear during childhood, often after the first year of life and frequently co-occur. Partial improvement of alopecia and nail dysplasia with JAK inhibitors has been described in isolated cases, suggesting a potential immune contribution (74). This constellation of clinical manifestations has been classified as DAVID syndrome—an acronym for deficient anterior pituitary with variable immune deficiency (70, 75). In addition, 66% of these patients were markedly susceptible to severe or recurrent viral diseases, and all of these patients were found to have detectable AAN-I-IFNs (27, 76). The most frequent diseases observed included life-threatening COVID-19 pneumonia, severe influenza, varicella, herpes zoster, and recurrent herpes labialis. Nine of these individuals suffered SARS-CoV-2 infection before vaccination: one (aged 23) died, and two (aged 17 and 41 years) required intensive care. This heightened viral susceptibility is strongly associated with the presence of AAN-I-IFNs, detected in over 80% of patients with C-terminal processing-resistant variants, supporting a causal role of these autoantibodies in the severity of viral diseases in these patients. These patients also displayed hypogammaglobulinemia and a progressive decline in B cell numbers, with particularly low proportions of memory B cells. Total T cell counts were generally normal, but counts of memory CD4^+^ T cells, Tregs, and Tfh cells were abnormally low (27, 68, 69, 77).

The second form of AD NF-κB2 disorders is caused by heterozygosity for truncating variants within the RHD domain (p.A35Efs*10, p.R52*, p.W270*, p.Y294Ifs*4, and p.K321Sfs*160)—the domain responsible for p52 dimerization and DNA binding—resulting in p52/p100 haploinsufficiency (Fig. 1 B). Eight individuals with p52/p100 haploinsufficiency have been reported to date (Table S1) (27, 78, 79). These variants underlie PAD with incomplete penetrance—including four cases of common variable immunodeficiency (CVID), one case of selective IgG2 subclass deficiency, and three asymptomatic individuals below 46 years of age—associated with autoimmune diseases in 50% of cases (autoimmune gastritis, lichen planus, celiac disease, and type 1 diabetes). Most symptomatic patients with p52/p100 haploinsufficiency had B cell lymphopenia, with normal or high proportions of switched memory B cells. Their T cell counts were normal, with low proportions of naïve CD4^+^ T cells and normal frequencies of Treg and Tfh cells (27, 78). The third form of AD NF-κB2 disorders is caused by heterozygosity for variants that truncate the ankyrin repeat domain of p100 (p.Q539*, p.R611*, and p.R635*), or just upstream (p.E418*), resulting in a gain of p52 activity (p52^GOF^) due to spontaneous nuclear translocation of the mutant proteins (Fig. 1 B) (80). Fifteen  patients from 7 kindreds have been reported to display PAD or CID with incomplete penetrance (50%, up to 48 years old) (81, 82, 83, 84) (Table S1). Patients have low to normal B cell counts, with abnormally low proportions of switched memory B cells. They have normal proportions of naïve CD4^+^ T cells, Treg, and Tfh cells and display normal T cell proliferation (27, 82). In marked contrast to patients with C-terminal processing-resistant p100 variants, none of the individuals with p52/p100 haploinsufficiency or p52^GOF^ variants studied to date has developed pituitary defects or ectodermal dysplasia. These patients were also not prone to the development of severe viral diseases, as attested by the asymptomatic or mild SARS-CoV-2 infections observed before vaccination in these two groups (27, 82). Furthermore, negative results were obtained for AAN-I-IFNs in all patients with p52/p100 haploinsufficiency or p52^GOF^ variants tested, consistent with their lack of severe viral disease.

**Figure 2.**
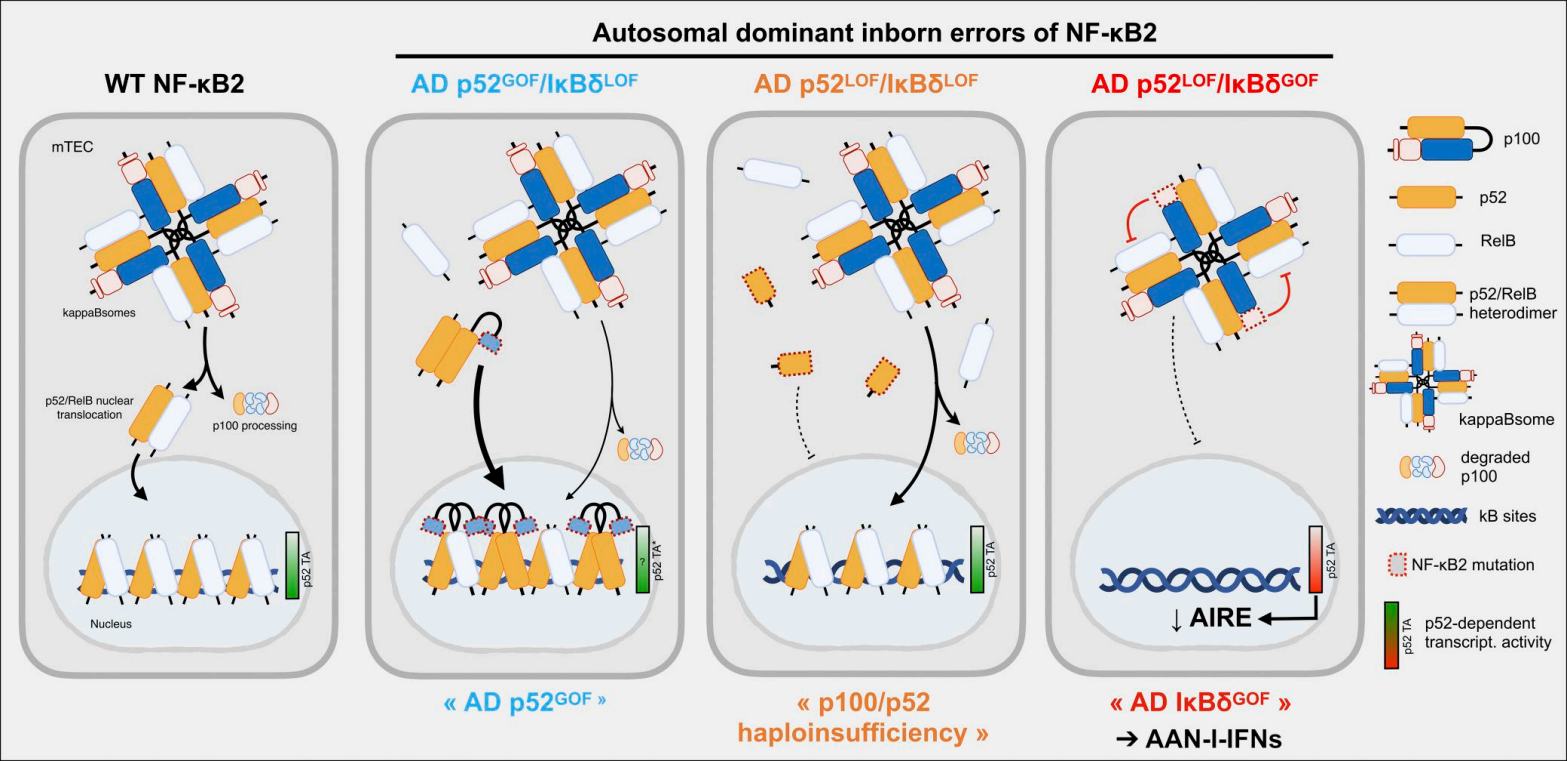
**Model illustrating the molecular consequences of the three forms of AD inborn errors of NF-κB2 in mTECs.** In the basal state, the NF-κB2 precursor p100 acts as a transcriptional inhibitor by forming cytoplasmic homomultimeric complexes. These complexes, known as “kappaBsomes,” bind p52 and RelB, restricting their transcriptional activity (referred to as the “IκBδ function” of p100). When the alternative NF-κB pathway is triggered by upstream TNF receptors (presumably RANK and/or CD40), the C-terminal part of the p100 precursor is processed into the proteasome to generate p52, the transcriptionally active form of NF-κB2, which dimerizes with RelB to form p52/RelB heterodimers. After translocation to the nucleus, this heterodimer binds to κB sites to regulate the transcription of targeted genes controlling mTEC development and functions, including *AIRE*. The p52^GOF^/IκBδ^LOF^ alleles are transcriptionally active but lack IκBδ function, leading to spontaneous nuclear translocation. In the heterozygous state, WT-p52 and mutant-p52 (if expressed) form normal or increased levels of transcriptionally active p52-containing homo- or heterodimers, leading to enhanced p52-dependent transcription. p52^LOF^/IκBδ^LOF^ alleles are non-functional for both p52 and IκBδ activities. In heterozygous cells (p52 haploinsufficiency), the pool of WT-p52– and RelB-dependent dimers is maintained at near-normal levels by transcriptional regulation and remains sufficient to support normal AIRE transcription and function. By contrast, p52^LOF^/IκBδ^GOF^ alleles are resistant to processing, resulting in a combination of impaired p52 generation, and high levels of IκBδ-dependent inhibitory activity of the mutant p100 against RelB and WT-p52. The amounts of WT-p52– and RelB-dependent dimers are markedly decreased, preventing sufficient AIRE-dependent central T cell tolerance to type I IFNs and leading to the development of AAN-I-IFNs. “p52 TA” reflects the level of p52-dependent transcriptional activity (TA), whether mediated by homodimers or heterodimers. For simplicity, only p52- homodimers or p52/RelB-containing dimers are represented. The expression of the proteins encoded by the p52^GOF^/IκBδ^LOF^ alleles has not been documented in patients’ cells.

These three defects have only recently been characterized precisely at the molecular level (27). Biochemical analyses of *NFKB2* alleles assessing both the p52-mediated transcriptional activity and the IκBδ-dependent inhibitory function have defined three distinct categories of mutants, as assessed by overexpression: (1) p52^LOF^/IκBδ^GOF^ variants, which are processing-resistant p100 variants that impair p52 production and trap RelB in the cytoplasm via the unprocessed p100; (2) p52^LOF^/IκBδ^LOF^ variants, which disrupt both p52-dependent transcription and IκBδ-mediated inhibition, resulting in a complete LOF; and (3) p52^GOF^/IκBδ^LOF^ variants, characterized by the constitutive translocation of truncated p52 mutant proteins into the nucleus (Fig. 2 and Table S3). The p52^LOF^/IκBδ^LOF^ variants are not expressed in T cells, fibroblasts, or monocyte-derived dendritic cells (MDDCs), resulting in almost normal nuclear translocation of WT p52/RelB heterodimer upon stimulation, causing p52/p100 haploinsufficiency (27, 78). The expression of the proteins encoded by the p52^GOF^/IκBδ^LOF^ alleles has not been documented in patients’ cells (81). Functional characterization of the fibroblasts of patients heterozygous for p52^LOF^/IκBδ^GOF^ variants revealed severe defects of p100 phosphorylation, p100 processing to generate p52, and the translocation of p52 into the nucleus after stimulation. This phenotype was observed in primary B cells, lymphoblastoid cell lines, or MDDCs stimulated with CD40L and in primary or SV40-immortalized fibroblasts stimulated with TWEAK or Lt, as in RelB- or NIK-deficient cells (24, 26, 27, 66, 85, 86). In addition, the accumulation of the unprocessed p100 mutants in the cytoplasm resulted in the formation of heteromultimeric complexes (κappaBsomes) sequestering RelB, WT-p100, and p52-containing complexes in the cytoplasm and interfering with the phosphorylation and subsequent processing of WT-p100 (22, 27). As a result, both p52 production and RelB activity were severely compromised, mirroring the defects seen in AR NIK deficiency. Among the three AD NF-κB2 IEIs, the p52^LOF^/IκBδ^GOF^ variants cause the most profound impairment of p52/RelB heterodimer activation in stromal cells, resembling the phenotype seen in AR IKK-α and NIK deficiency.

### AD TRAF3 deficiencies

TRAF3 acts, together with TRAF2 and the E3 ubiquitin ligases cellular inhibitor of apoptosis 1 (cIAP1) and cIAP2, as a negative regulator that constitutively degrades NIK, thereby preventing spontaneous activation of the alternative NF-κB pathway under resting conditions (4). TRAF3 also functions downstream from the retinoic acid-inducible gene I-like receptors (RLRs) and the Toll-like receptors (TLRs), such as TLR3, by mediating type I and type III IFN induction (87). AD TRAF3 deficiency was first reported in 2010 in a patient with herpes simplex virus encephalitis (HSE) heterozygous for the p.R118W *TRAF3* variant, which is located within the zinc-finger domain (28). The R118W allele has dominant-negative activity, associated with impaired TRAF3-dependent TLR3 and cytosolic dsRNA sensor responses *in vitro*. Individuals heterozygous for the R118W allele have an abnormally small proportion of MZ B cells and a high proportion of CD27^+^CD38^+^ antibody-secreting cells (ASCs) (28, 29). The p.R118W variant is expressed at levels half those for the control, and heterozygous B cells from patients produce larger amounts of p52 than control cells (28, 29). Twelve patients from 7 unrelated kindreds heterozygous for truncating variants of *TRAF3* have been reported (29, 88) (Table S1). They were heterozygous for five different nonsense or frameshift *TRAF3* variants (p.Q114*, p.R163*, p.S356Pfs*6, p.Q407*, and p.Y425*). These genetic variants were inherited from affected parents or occurred *de novo*, with complete clinical penetrance. The patients suffered from recurrent ear and sinopulmonary diseases due to encapsulated bacteria, including *Streptococcus pneumoniae* and *Haemophilus influenzae*, beginning in childhood. They also displayed lymphoproliferative diseases (e.g., lymphadenopathy and splenomegaly), autoimmunity (Sjögren’s syndrome and Hashimoto’s thyroiditis), enteropathy, or atopic disorders (food and drug allergies and high levels of IgE). None had a history of HSE. Pathology analyses of the gastrointestinal tract, lungs, lymph nodes, or spleen have revealed lymphoid hyperplasia with enlarged GCs or granulomatous formations. Most patients display hypergammaglobulinemia, with high levels of IgG and IgM and variable levels of IgA, but four patients developed CVID in adulthood and presented panhypogammaglobulinemia (29, 88). Regardless of their IgG levels, all patients had impaired responses to polysaccharide vaccines. Total B cell counts were normal, but patients had high naïve B cell counts and markedly smaller than normal proportion of class-switched memory B cells, together with normal to high proportions of CD21^low^ B cells and ASCs. Their T cell compartments contained abnormally small numbers of naïve CD4^+^ and CD8^+^ T cells (with a corresponding increase in the proportions of memory T cells), with normal T cell proliferation in response to anti-CD3/CD28 antibody stimulation. The patients had high proportions of Tregs and Tfh cells (29).

PBMCs from patients with truncating *TRAF3* variants displayed markedly low levels of TRAF3 expression (∼20% control levels), which could be increased to 50% control levels by treatment with the proteasome inhibitor MG132 (29). This suggests that proteasomal degradation of the WT TRAF3 allele is enhanced at baseline, probably due to a low TRAF3-to-TRAF2-cIAP1/2 ratio. Patient-derived EBV-transformed B cells displayed a spontaneous increase in NIK expression, p100 phosphorylation, and p100 processing to p52, along with an upregulation of *MAP3K14*, *CHUK*, *NFKB2*, and *RELB* transcript levels, indicative of constitutive NF-κB2 activation (29). Consequently, B cells from patients with truncating TRAF3 variants displayed enhanced responses to BAFF, which predominantly activates the BAFFR-dependent alternative NF-κB pathway in B cells (29), whereas CD40 and BCR signaling primarily engage the canonical NF-κB pathway in B cells. The *in vitro* response of the patients’ B cells to CD40L plus IL-21 was normal in terms of plasmablast generation and IgG secretion. B cells heterozygous for a TRAF3-truncating variant displayed a spontaneous upregulation of transcript levels for *TRAF6* and *REL*—two NF-κB proteins essential for CD40-mediated signaling—*ex vivo*, along with an enhanced response to anti-IgM/IL-4 or TLR9 stimulation *in vitro. *These observations suggest that human TRAF3 plays a crucial role in downregulating NIK/p52-dependent alternative NF-κB pathway activation and in modulating T-independent (TI) Ag responses, probably via the regulation of TLR9-dependent, BCR-proximal, and possibly CD40-dependent signaling in human B cells (89).

## Inborn errors of TNFRs and TNFR ligands driving activation of the alternative NF-κB pathways

### AR BAFFR deficiency

BAFFR is expressed exclusively on B cells, at levels varying according to B cell maturation stage (from transitional to mature B cells) (Table 1) (90). Unlike TACI and BCMA, which can bind both BAFF and APRIL, BAFFR binds only BAFF (91). AR complete BAFFR deficiency has been reported in two siblings from a consanguineous family (92). They were homozygous for an in-frame p.L89_V96del *TNFRSF13C* variant removing eight amino acids from the transmembrane region. Both patients suffered from recurrent respiratory tract infections caused by encapsulated bacteria (*S. pneumoniae* and *H. influenzae*), and one developed severe shingles at 70 years of age. The immunological phenotype observed in patients with AR BAFFR deficiency closely resembles that of *Baffr*-KO mice (5, 6) (Table S2). Serum IgM and IgG levels are low, IgA levels are normal, and there is a prominent population of IgA^+^ plasma cells in the gut. Despite multiple *S. pneumoniae* infections, the patients were unable to mount an TI antibody response following pneumococcal polysaccharide vaccination, whereas their TD antigen response was unaffected. Patient B cell counts were very low, with a developmental block at the transitional stage, resulting in a strong decrease in counts of MZ B cells and low proportions of class-switched memory B cells. Furthermore, the patients’ CD27^+^ B cells had lower levels of TACI than control cells, suggesting that BAFFR signaling regulates TACI expression in CD27^+^ B cells. The p.L89_V96del *TNFRSF13C* variant was shown to be a loss-of-expression in patient CD19^+^ B cells or EBV–B cell lines and LOF, losing its ability to bind BAFF (92). Overall, these data underscore the crucial role of BAFFR signaling in the development of mature peripheral B cells beyond the transitional B cell stage, as demonstrated by the significantly lower number of peripheral B cells in patients than in age-matched controls, specifically affecting all mature B cell subsets without affecting the transitional subsets. This developmental arrest after the transitional cell stage strongly suggests that human BAFFR is essential for the survival of transitional B cells and their differentiation into follicular and MZ B cells.

### XL-CD40L in AR CD40 deficiency

CD40L is a transmembrane glycoprotein expressed principally on activated T cells. It binds to the CD40 present on B cells and various other cell subsets, including antigen-presenting cells (APCs), such as dendritic cells and monocytes/macrophages, as well as endothelial cells, epithelial cells, and stromal cells, such as mTECs (Table 1) (93). CD40 signaling induces both the canonical and alternative NF-κB pathways (94). CD40L deficiency, first reported in 1993, causes an XL CID characterized by defects of both humoral immunity—due to impaired CSR and SHM—and cellular immunity, affecting APC–T cell interactions (95, 96). This condition is commonly referred to as XL hyper-IgM (XL-HIGM) syndrome (95). More than 200 patients with XL-CD40L deficiency have been reported worldwide, and many more have been diagnosed, as this is a relatively common IEI. More than 100 variants of *CD40LG* have been reported, most of which are frameshift deletions, splicing, nonsense, or missense variants affecting the extracellular domain of CD40L (93). These variants typically result in the loss of protein expression and/or impairment of the interaction with CD40. The disease classically presents within the first year of life, characterized by recurrent sinopulmonary tract infections caused by encapsulated bacteria and heightened susceptibility to severe *Pneumocystis jirovecii* and *Cryptosporidium* spp. infections, and, more rarely, to *T. marneffei* or *Cryptococcus* spp. infections (97). In some cases, hypomorphic variants of *CD40LG* present with atypical clinical features, including a later onset and a less severe clinical course than classical XL-HIGM syndrome (46). Despite the occurrence of severe viral diseases (including diseases due to CMV, enterovirus, or RSV) in some patients, no AAN-I-IFNs were detected, even in adult patients who had not undergone HSCT (27). More than half the patients display neutropenia.

There are no GCs in the lymph nodes of XL-CD40L-deficient patients. IgG, IgA, and IgE are undetectable or present at very low levels, whereas IgM levels are normal or high, and patients have a poor response to TD antigens due to impaired CSR. B cell counts are normal, but CD27^+^ switch memory B cells are absent or present at extremely low levels (46). T cell numbers and proliferation in response to mitogens remain normal. AR CD40 deficiency, first described in 2001, causes an HIGM syndrome clinically and immunologically indistinguishable from XL CD40L deficiency, with fewer than 50 cases reported worldwide to date (98). Twenty-eight variants of *CD40* have been reported, most of which affect essential splicing sites and impair CD40 expression on B cells (99). These phenotypes are consistent with those of *Cd40lg*- or *Cd40*-KO mice, which display normal thymic and SLO development but lack GC formation and have an impaired TD antigen response (Table S2) (7, 8). Together, these data demonstrate that CD40L/CD40 activation is essential for GC formation and antibody isotype switching and that SHM is required for antigen affinity maturation and to support the generation of memory B cells *in vivo*.

### AR LTβR deficiency

LTβR is mostly expressed on stromal cells, including lymph node stromal cells (Table 1) (13). It binds LTα1β2, a ligand specific for LTβR (100), and LIGHT (TNFSF14), which also binds LIGHT-R/HVEM (101). These ligands are mostly expressed on activated lymphocytes (13). LTβR activates both the canonical and noncanonical NF-κB pathways, but with a stronger effect on the noncanonical pathway (102). AR complete LTβR deficiency has recently been reported in three patients from two unrelated consanguineous Turkish families (100). These patients were homozygous for the p.Q31* or p.R120P variants located in the extracellular domain of LTβR. The clinical manifestations observed in these patients included early-onset recurrent upper and lower respiratory tract infections, predominantly of bacterial origin. One patient developed *S. pneumoniae* meningitis, another had an episode of acute hepatitis with biliary destruction, and an older brother with similar disease manifestations died from complications. One patient suffered from flat warts, but none developed overt autoimmune diseases, unlike *Ltbr*-KO mice (Table S2) (9). Lymphoscintigraphy revealed lymph node aplasia and an absence of tonsils despite recurrent infections. The spleen was of normal size and morphology, but the presence of Howell–Jolly bodies in peripheral blood smears indicated functional asplenia. This is consistent with the findings for *Ltbr*-KO mice, which lack peripheral lymph nodes and Peyer’s patches (9). Patients had low levels of circulating IgG and IgA, with low to normal IgM levels. They had normal ranges of total leukocyte and lymphocyte counts. Despite their normal counts of total CD19^+^ cells, the patients had low levels of GC-like B cells and an almost total absence of class-switched and unswitched memory B cells, and of IgA^+^ or IgG^+^ B cells, with normal numbers of T-bet^high^CD21^low^ B cells. However, upon* in vitro* stimulation with CD40L and IL-4 or IL-21, B cells showed normal CD40L-dependent activation and proliferation and were able to undergo class switching to IgA- or IgG-positive cells. TREC levels were lower than those in age-matched controls, but the proportions of RTE and naïve CD4^+^ T cells and the total numbers of T cells and CD4^+^ T cells were normal. However, a lower level of TCR diversity was observed.

*In vitro*, T cell proliferation upon mitogen activation was normal. The proportions of Tregs and Tfh cells were low (100). The preserved functions of the patients’ lymphocytes *in vitro*, despite impaired differentiation *in vivo*, suggest that the defects are not intrinsic to the lymphocytes, instead stemming from alterations to the stromal compartments and the SLOs. No AAN-I-IFNs were detected in the single patient tested, consistent with the normal Aire expression observed in *Ltbr*-KO mice (103, 104). The primary fibroblasts of the patients displayed no LTβR expression and failed to upregulate p52 after LTα1β2 stimulation, both these defects being corrected by genetic rescue. B cell development was normal up to the naïve B cell stage, as was B cell repertoire diversity, but the proportions of GC and memory B cells were low, as were levels of SHM, suggesting a defect of the GC reaction. Consistent with the role of LTβR signaling in the development and organization of SLOs, the levels of CXCL13—a chemokine produced by GC stromal cells under the control of LTβR—in patient plasma were low. When cocultured with activated MDDCs and stromal cells to reconstitute the GC architecture *ex vivo*, patient B cells differentiated into CD27^+^ memory B cells and upregulated activation-induced cytidine deaminase (AID), the enzyme that initiates SHM. Human LTβR signaling is, thus, essential for SLO development and stroma-intrinsic terminal B cell maturation in the GC. However, it appears to be redundant for AIRE-dependent central tolerance.

### AR RANK and RANKL deficiencies

RANK (*TNFRSF11A*), which is expressed on cells of the myeloid lineage, including osteoclasts, interacts with RANKL (*TNFSF11*), which is expressed on cells of the mesenchymal lineage (osteoblasts) in bones. AR RANKL deficiency has been reported in nine patients from seven unrelated kindreds suffering from osteopetrosis caused by homozygosity for variants of the *TNFSF11* gene predicted to be deleterious (105, 106). AR RANK deficiency has been reported in 23 patients with osteopetrosis from 14 kindreds homozygous or compound heterozygous for 17 variants of the *TNFRSF11A* gene (107). Patients with both conditions present early-onset osteoclast-poor osteopetrosis, characterized by dense bones and osteosclerosing dysplasia leading to fractures, visual impairment, and neurological defects, with an absence of osteoclasts in bone tissues. They lack palpable lymph nodes and occasionally suffer from hepatomegaly and/or recurrent respiratory tract infections (107). Some RANK-deficient patients display mild hypogammaglobulinemia, a lack of antibody response to tetanus toxoid vaccination, with low proportions of IgD^−^CD27^+^ memory B cells in the four cases evaluated. Osteopetrosis is occasionally observed in patients with XL-NEMO deficiency (OL-EDA-ID, osteopetrosis, lymphedema, hypohidrotic ectodermal dysplasia, and immunodeficiency), but not in patients with inborn errors of IKK-α, NIK, RelB, or NF-κB2. This suggests that RANK signaling operates, at least in part, through the canonical NF-κB pathway to support osteoclast development and function. HSCT remains the only curative treatment for RANK deficiency. However, it has no beneficial effect on disease progression in patients with AR RANKL deficiency, due to the stromal origin of the defect, with insufficient donor stromal precursors or mesenchymal stem cells. These observations highlight the essential nature of the RANK–RANKL axis for osteoclast differentiation, with an apparent redundancy in humoral immunity. They also suggest that NF-κB2 and RelB are functionally redundant for osteoclast differentiation in humans. In mice, Rank controls the development of Aire^+^ mTECs (10). The role of the RANKL-RANK axis in AIRE^+^ mTEC maturation and the consequences of its deficiency in terms of AAN-I-IFN development remain unknown in humans.

## Conclusions and perspectives

Inborn errors of the core components of the alternative NF-κB pathway (NIK, IKK-α, RelB, and NF-κB2), their negative regulator (TRAF3), four related TNFRs (BAFFR, CD40, RANK, and LTβR), and two of their ligands (CD40L and RANKL) have been described in humans (Table 2). The corresponding mouse models generally display a similar phenotype. Mice deficient for *Ltbr*, *Map3k14*, *Chuk*, *Relb*, or *Nfkb2* display impaired GC development (43, 65, 108, 109), whereas mice deficient for *Baffr*, *Map3k14*, *Relb*, or *Nfkb2* display various degrees of B cell lymphopenia (43, 65, 110, 111, 112). These models closely mirror the SLO hypoplasia and humoral immunodeficiency reported in human inborn errors of *LTBR* and *BAFFR*, respectively, and in human inborn errors of *MAP3K14*, *CHUK*, *RELB*, and *NFKB2*. Furthermore, *Map3k14*-, *Chuk*-, *Relb*-, *Nfkb2*, and *Rank*-deficient mice display defective mTEC development, leading to impaired Aire-dependent central T cell tolerance and organ-specific autoimmunity (10, 40, 113, 114). These defects are mTEC intrinsic, as further confirmed by studies of mTEC-specific KO models targeting *Map3k14*, *Chuk*, and *Relb* (39, 115). This finding is consistent with the development of AAN-I-IFNs in patients with inborn errors of NIK, IKK-α, RelB, and NF-κB2, in whom p52/RelB heterodimer activity is severely disrupted. Conversely, mouse models with constitutive activation of the alternative NF-κB pathway (*Traf3*-KO) in B cells display SLO hyperplasia and enhanced B cell survival (116, 117, 118, 119, 120), mirroring features observed in patients with AD TRAF3 haploinsufficiency.

Despite the significant contribution of animal models to our understanding of the essential role of the alternative NF-κB pathway, several limitations can be highlighted, concerning, in particular, the specific transcriptional activity of p52, one of the central components of the NF-κB pathway. Since its initial cloning in 1991 (121), most studies aiming to elucidate the function of NF-κB2 in mice have been based on complete or conditional *Nfkb2*-KO models. These models display an effective abolition of p52-dependent transcriptional activity, but they also present a simultaneous elimination of the IκBδ inhibitory regulator, potentially affecting the transcription of a broader range of targeted genes through release of the cytoplasmic retention of cRel, RelA, or RelB (22, 23, 122). This dual disruption makes it difficult to determine the specific contribution of the p52-dependent heterodimer, thereby hindering a comprehensive understanding of its essential role (109, 112, 123). Mouse models with a selective abolition of IκBδ function (Nfkb2^ΔCT/ΔCT^, corresponding to human p52^GOF^/IκBδ^LOF^) (124), or a selective increase in this activity due to a p100 processing-resistant mutant (*Lym1* mouse, p52^LOF^/IκBδ^GOF^) (114, 125), have helped to distinguish between these two functions. However, there is still no mouse model in which p52 function is specifically abolished with no effect on IκBδ regulatory function (p52^LOF^/IκBδ^WT^).

Experiments in mice have also revealed key immunological differences, even in knock-in models accurately reconstructing the human allelic variants (126). Studies of mouse models of p52^LOF^/IκBδ^GOF^ human mutants and of *Relb*-, *Nik*-, and *Ikka*-deficient mice, have confirmed the mTEC-intrinsic defect in central tolerance, characterized by lower levels of medulla formation, mTEC developmental defects, and impaired AIRE expression (27, 40, 113, 114, 125). However, despite the development in these mice of severe systemic autoimmunity with T cell infiltration, contrary to humans with the equivalent defect, these mice do not produce AAN-I-IFNs (27, 114, 125). This finding is reminiscent of those for mouse models of APS-1, such as *Aire*-KO mice, which fail to develop autoantibodies against type I IFNs (127, 128), whereas such autoantibodies are detected in *Aire*-KO rat models, regardless of genetic background (129, 130). However, the immunological and clinical phenotypes of patients with inborn errors of the alternative NF-κB pathway may differ significantly from those of the corresponding mutant mice. For example, mice heterozygous for a null *Nfkb2* allele (*Nfkb2*^+/−^) or for an *Nfkb2* C-terminal deletion (*Nfkb2*^+/ΔCT^) have no overt immune phenotype, unlike patients with AD p52/p100 haploinsufficiency or AD p52^GOF^ (27, 78, 114). Similarly, mouse models of human p52^LOF^/IκBδ^GOF^ mutants do not develop the ectodermal and pituitary defects observed in about 25% of human patients (114, 125), making it difficult to determine whether these features result from intrinsic developmental defects or from the impact of leukocytic abnormalities (74, 131). Conversely, mouse models have been instrumental in analyses of the role of the alternative NF-κB pathway in cells and tissues not accessible in human patients. In particular, they have made a significant contribution to our understanding of the stroma- versus hematopoietic cell-intrinsic roles of most components of this pathway through adoptive transfer experiments and conditional KO models (Table S2).

The description of inborn errors of the alternative NF-κB pathway has helped to reveal the essential role of this pathway in human immunity (1, 30). No case of human AR complete NF-κB2 deficiency (p52^LOF^/IκBδ^LOF^) has yet been reported. Identifying such a defect, or a defect selectively abolishing p52 function (AR p52^LOF^/IκBδ^WT^), would provide insight into the nonredundant role of p52-dependent dimers involving RelB or other NF-κB transcription factors, including p52 itself. The identification of such a defects would therefore help to determine whether the clinical manifestations observed in patients with AD p52^LOF^/IκBδ^GOF^ variants, such as ectodermal and pituitary defects, and the production of AAN-I-IFNs are due exclusively to the loss of p52-dependent transcriptional activity or whether they are due to enhanced IκBδ inhibitory effects on other NF-κB2 binding partners. In addition, the discovery of other allelic forms of inborn errors at known loci, such as GOF variants of *MAP3K14*, *RELB*, or *CHUK*, or AR complete TRAF3 deficiency (if not embryo-lethal in humans), would shed additional light on the mechanisms regulating the alternative NF-κB pathway. The identification of IEIs affecting adaptor proteins regulating TNFR signaling, such as TRAF6 or TRAF2, regulators, such as cIAP1 or cIAP2, and TNFRs, such as TNFR2—all previously implicated in p52/RelB signaling—will be crucial to decipher the essential, cell-intrinsic roles of these components, their upstream receptors, and ligands in controlling p52/RelB heterodimer activity. For example, the identification of patients with deficiencies of LTα_1_β_2_ or LIGHT (both binding to LTβR), or BAFF (which can bind BAFFR, TACI, and BCMA), would provide insight into the essential or redundant roles of these ligands in stromal and hematopoietic human immunity, respectively. In addition, the TNFRs and corresponding ligands controlling AIRE-dependent maturation and the development of AAN-I-IFNs remain to be discovered. Finally, identifying autoimmune phenocopies of these IEIs due to neutralizing autoantibodies against circulating cytokines (such as soluble CD40L, Lt, or BAFF) would also shed light on the clinical consequences of the corresponding deficiencies.

An understanding of the molecular mechanisms underlying the immunological and clinical phenotypes of patients is essential to guide therapeutic interventions. The stroma-intrinsic defects of patients with inborn errors of the alternative NF-κB pathway render them vulnerable to severe viral diseases throughout life, due to the persistence of AAN-I-IFNs, and possibly other autoimmune diseases resulting from defective central tolerance (26, 27). Therefore, while HSCT can cure the hematopoietic defect causing CID in patients with AR IKK-α, NIK, or RelB deficiency, this procedure is associated with very high post-transplant mortality in patients with AD p52^LOF^/IκBδ^GOF^ disorder (Table S1) (24, 27, 60). Moreover, stromal-intrinsic defects affecting lymphoid organogenesis in these patients may compromise engraftment, limit immune reconstitution, and adversely affect HSCT outcome (60). For some of these patients, alternative strategies, such as allogeneic thymus implantation or the development of induced pluripotent stem cell -derived mTECs may be possible options for treatment (132, 133). Finally, as about 30% of patients with B cell or plasma cell neoplasia carry somatic variants, somatic copy number variations, or translocations of genes from the alternative NF-κB pathway (*NFKB2*, *MAP3K14*, or *TRAF3* loci) (134), pharmacological interventions targeting this pathway, such as small molecules inhibiting NIK (NIK inhibitors) or inactivating cIAPs (SMAC mimetics), may open up promising new avenues for treatment in patients with inborn errors of the alternative NF-κB pathway.

## Online supplemental material

Table S1 shows clinical and immunological features of patients with inborn errors of the “core” proteins of the alternative NF-κB pathway. Table S2 shows main features of mouse models of inborn errors of the alternative NF-κB pathway. Table S3 shows *NFKB2* variant nomenclature and functional consequences.

## Supplementary Material

Table S1 shows clinical and immunological features of patients with inborn errors of the “core” proteins of the alternative NF-κB pathway.

Table S2 shows main features of mouse models of inborn errors of the alternative NF-κB pathway.

Table S3 shows *NFKB2* variant nomenclature and functional consequences.
