# Supplementary material for: Human inborn errors of the alternative NF-κB pathway
Source: J Hum Immun. 2025 Nov 21;2(1):e20250104. doi: 10.70962/jhi.20250104 (PMC12829755; doi:10.70962/jhi.20250104)
Supplement: Table S1 — shows clinical and immunological features of patients with inborn errors of the “core” proteins of the alternative NF-κB pathway. [file jhi_20250104_tables1.docx]

| Gene | IEI | Number of patients | Mean age at onset of immunodeficiency [range] | Type of IEI (penetrance) | Bacterial infections | Fungal infections | Mycobacterial infections | Parasitic infestations | Viral (severe or recurrent) | Auto-Abs against  type I IFNs | Alopecia aerata/totalis (age at onset ±SD, range) | Trachonychia (age at onset ±SD, range) | Other ectodermal dysplasia features* | Autoimmunity / organ T-cell infiltration | Lymphoproliferation | Other | Hypo-IgM | Hypo-IgG | Hypo-IgA | Low B cell counts | Low % smB cells | Low T-cell counts | Low proportion of memory CD4+ T cells (%) | Tregs/  TFh | HSCT  (n=survivors  /total) | References |
| --- | --- | --- | --- | --- | --- | --- | --- | --- | --- | --- | --- | --- | --- | --- | --- | --- | --- | --- | --- | --- | --- | --- | --- | --- | --- | --- |
| *NFKB2* | AD p52^LOF^/IκBδ^LOF^  (p52/p100 haploinsufficiency) | 8 | 26 [18-33] | PAD (63%) | Yes (RTI, 50%) | NR | NR | NR | Herpes labialis, *n*=1 | No | NR | NR | NR | Yes (50%) | NR | - | 42% | 42% | 42% | 33% | 33% | 14% | 33% | =/↓ | No | (143, 141, 144) |
|  | AD p52^GOF^/IκBδ^LOF^ | 15 | 10 [5-14] | PAD (50%) | Yes (RTI, 50%) | CMC, *n*=1 | NR | NR | Yes (*n*=2, 13%: HSV, CMV, EBV, warts) | No | NR | NR | NR | Hypothyroidism (*n*=1) | Yes (20%, splenomegaly, lymphadenopathy) | - | 45% | 42% | 63% | 40% | 15% | 30% | 23% | =/= | No |  |
|  | AD p52^LOF^/IκBδ^GOF^  (DAVID syndrome) | 107 | 4 [1-31] | PAD (98%) | Yes (RTI, >90%) | 15% | NR | NR | Yes (*n*=71, 66%: SARS-CoV-2, severe influenza, HSV) | Yes (82%) | Yes (30%) (6,0±4,3 years, 1-14) | Yes (25%) (4,8±4,1 years, 1-10) | Yes (3,7%) | Yes (28%, mostly hypothyroidism, psoriasis, vitiligo) | <5% | Pituitary endocrine defects (38%) | 84% | 86% | 100% | 71% | >90% | <10% | >50% | ↓/↓ | Yes (n=1/4) |  |
| *RELB* | AR RelB deficiency | 9 | 1 [1-3] | CID (100%) | Yes (RTI: 77%; *Salmonella* spp., *n*=1) | Yes (22%: CMC, *Talaromyces marneffei*, *Cryptococcus neoformans)* | Yes (*MTb,* *n*=1) | NR | Yes (44%: HSV-1, VZV, adenovirus, epidermodysplasia verruciformis, JC polyomavirus) | Yes (88%) | NR | NR | NR | Yes (33%: dermatitis, hepatitis, enteropathy, primary sclerosing cholangitis) | DLBCL (*n*=1) | - |  | 44% |  | 44% | 44% | 22% | Low levels of naïve T cells in 44% | =/= | Yes (n=4/4, mixed chimerism in 2) | (142, 149, 140, 145) |
| *CHUK* | AR deficiency IKK-α (excluding Cocoon syndrome) | 7 | 1 [1-2] | CID (100%) | Yes (RTI, 100%; *Salmonella* spp., *n*=2) | Yes (CMC, 50%) | NR | NR | Yes (100%: rotavirus, coxsackie, RSV, SARS-CoV-2, adenovirus, bocavirus, rhinovirus, CMV; epidermodysplasia verruciformis) | Yes (100%) | Yes (42%) (at birth) | Yes (20%) (at birth) | Yes (42%) (at birth) | Yes (33%, hepatitis or enteropathy) | Lymph node hypoplasia (100%); hepatosplenomegaly, 33%; DLBCL (*n*=1) | Skeletal malformations, cleft palate | 83% | 100% | 83% | 66% | 100% | NR | 20% | ↓/↓ | No | (139, 147, 136, 138) |
| *MAP3K14* | AR NIK deficiency | 8 | 1 [1-2] | CID (100%) | Yes (RTI, 88%) | Yes (75%, CMC) | Yes (37%, BCG-osis) | Cryptosporidium (25%) | Yes (50%, CMV, norovirus) | Yes (100%) | NR | NR | NR | NR | Lymph node hypoplasia | - | 88% | 88% | 100% | 50% | 100% | NR | 66% | =/↓ | Yes (n=2/4) | (151, 143, 137, 148) |
| *TRAF3* | AD TRAF3 haploinsufficiency | 12 | Childhood | Immune dysregulation | Yes (RTI, 100%) | NR | NTM (*n*=1) | NR | NR | No | NR | NR | NR | Yes (Sjögren’s syndrome, thyroiditis, enteropathy) | Yes (75%, splenomegaly, lymphadenopathy) | Allergies, high IgE levels (25%) | NR | 33%; (hyper-IgG in 58%) | NR | 8% | 100% | 90% | High memory CD4+ T-cell levels in 100% | ↑/↑ | No | (146, 150) |

**Supplementary Table I: Clinical and immunological features of patients with inborn errors of the “core” proteins of the alternative NF-κB pathway**

*Ectodermal dysplasia features other than alopecia and trachonychia; include early onset sparse body hair, eyebrows or eyelashes, hypohidrosis, or dental anomalies (microdontia).

CMC: chronic mucocutaneous candidiasis

HSCT: hematopoietic stem cell transplantation

NTM: non-tuberculous mycobacteria

NR: not reported
