# Supplementary material for: Human inborn errors of the alternative NF-κB pathway
Source: J Hum Immun. 2025 Nov 21;2(1):e20250104. doi: 10.70962/jhi.20250104 (PMC12829755; doi:10.70962/jhi.20250104)
Supplement: Table S2 — shows main features of mouse models of inborn errors of the alternative NF-κB pathway. [file jhi_20250104_tables2.docx]

**Supplementary Table II: Mouse models deficient for components of the alternative NF-kB pathway**

| Gene | Mouse model | Corresponding IEI | Mouse model with genetic background | Viability & lifespan | Skeletal/epidermal malformations | Tissue infiltration | Thymus | Secondary lymphoid organs | B-cell lineage (maturation/survival) | Humoral immunity | T-cell lineage | Dendritic cells | Other features | MGI  Accession number (online references#) |
| --- | --- | --- | --- | --- | --- | --- | --- | --- | --- | --- | --- | --- | --- | --- |
| *NFKB2* | p52^-/-^p100^-/-^ | Not reported | *Nfkb2^xdr/xdr^* (xander)  BALB/c  C57BL/6  Nfkb2^tm2Brv^  C57BL/6  Nfkb2^tm1Sbn^  C57BL/6 | Viable, normal to near-normal** | No | Absent (4 weeks) to mild T-cell infiltration** (4-5 months): lung, liver, salivary glands, kidneys, stomach with anti-tissue auto-Abs (§) | Normal thymic architecture  Low to normal numbers of UEA-1+ mTEC**,  **Normal Aire expression**** | incomplete LN development, lack PP (§)  reduced B cell follicles, no MZ in spleen (§)  Impaired secondary GC structure and mature FDC network formation in spleen and LN (§) | Normal B-cell development in the BM  Slightly low numbers of cells for peripheral, LN, and splenic B-cell lineages (¶, B cell)  **Low proportions and numbers of cells for splenic B-cell lineages** (including T1, T2, MZ and follicular) (¶, B cell) | Low basal levels of IgG3 and IgA  **impaired Ab response to TD antigens****, normal to impaired responses to type 1 TI and type 2 TI antigens** | Normal (thymic) or modest increased (splenic and LN) numbers of Tregs (¶, Treg)  Impaired thymic iNKT development (§) | Normal thymic and splenic DC numbers | - | [MGI:2667230](https://www.informatics.jax.org/allele/MGI:2667230)  [MGI:2180306](https://www.informatics.jax.org/allele/https:/www.informatics.jax.org/allele/MGI:2667230)  [MGI:2179705](https://www.informatics.jax.org/allele/MGI:2179705)  [MGI:2675476](https://www.informatics.jax.org/allele/MGI:2675476) |
|  | p52^-/+^p100^-/+^ | p100/p52 haploinsufficiency  (AD p52^LOF^/IκBδ^LOF^) | *Nfkb2^xdr/+^* (xander)  BALB/c  C57BL/6 | NA | No | NA | NA | NA | Normal numbers of peripheral and splenic mature B cells | Slightly impaired Ab response to TD antigens; normal responses to TI antigens | Normal T cell development | NA | - | [MGI:2667230](https://www.informatics.jax.org/allele/MGI:2667230) |
|  | p52^+/+^p100^-/-^ (Nfkb2^ΔCT/ΔCT^) = 451* | Not reported | Nfkb2^tm1Brv^  C57BL/6 | Impaired: death 3 to 4 weeks after birth (gastric hyperplasia) | Osteopenia, ossification maturation defect with dwarfism and shortened long bones | Severe T-cell and macrophage infiltration: lungs, salivary glands, lamina propria (¶) | Thymic atrophy | Enlarged LN with increased paracortical areas,  reduced spleen size with abnormal architecture | Impaired BM B-cell development (pre–pro-B to pro–B-cell stage) (¶)  small number of splenic immature and transitional cells, **increase in the proportion of MZ B cells in spleen** (¶) | Low basal IgG3 and high IgA levels (¶)  impaired Ab response to TI antigens (¶, B-cell) | Increased numbers of peripheral T cells  Normal thymic Treg proportions, decreased Tregs proportions in the spleen and peripheral LN | Increased numbers of BM and peripheral myeloid cells  Reduced cDC2 numbers in the spleen | **Severe gastric hyperplasia**  large numbers of granulocytes in the BM and blood | [MGI:2179689](https://www.informatics.jax.org/allele/MGI:2179689) |
|  | p52^-/+^p100^-/+^ (Nfkb2^ΔCT/+^) | AD p52^GOF^/IκBδ^LOF^ | Nfkb2^tm1Brv^  C57BL/6 | Normal | No | NA | NA | NA | NA | NA | NA | NA | mild gastric hyperplasia | [MGI:2179689](https://www.informatics.jax.org/allele/MGI:2179689) |
|  | p52^+/-^p100^+/GOF^ (Nfkb2^+/Lym1(Y868*)^) | DAVID syndrome (AD p52^LOF^/IκBδ^GOF^) | Nfkb2^D865G/+^  Nfkb2^Y868indel/+^  Nfkb2^S866fs/+^  C57BL/6  Nfkb2^Lym1/+^  BALB/c  C57BL/6 | almost normal to markedly reduced** | No | Mild to severe T-cell infiltration: pancreas, lacrimal and salivary glands, liver, lung, ear canal and prepuce (§, stroma-dependent) | **Thymic medullary dysplasia**  **low number of UEA1^+^ mTECs**  **reduced Aire expression** | **Absence of some peripheral LN and PP**  reduced follicles and FDC network in LN  Reduced MZ formation in spleen | Normal B-cell development in BM (hom*)  B-cell lymphopenia in the spleen  Mild to severe decrease in the number of peripheral mature B cells post transitional T_2_ stage (hom*) | Low levels of IgM, IgG1, IgG2b, and IgA (hom*) | Decreased thymic and splenic Treg numbers and proportions (¶ and §) | NA | Peripheral T- and B-cell lymphocytosis (hom*), vitiligo, facial and ear dermatitis | [MGI:4412046](https://www.informatics.jax.org/allele/MGI:4412046) |
| *RELB* | Relb^-/-^ | AR RelB deficiency | Relb^tm1Brv^  C57BL/6  [Relb^Tg(H2-K1/GH1)106Bri^](https://www.informatics.jax.org/allele/MGI:2179544)  C57BL/6 | Impaired: death from 7–8 weeks to several months of age (inflammatory disease) | No | Severe T-cell infiltration: lung, liver, salivary glands, skeletal musculature, gastrointestinal tract, stomach, epididymis and anti-tissue auto-Abs (§, mTEC) | **Thymic atrophy, no medulla formation,**  **very low UEA-1^+^ mTEC numbers**  **no Aire expression** | **Incomplete LN development (§), lack of PP**  Absent B cell follicle formation and FDC network  no splenic MZ formation (¶) | Normal to slightly low mature B-cell counts in the spleen and periphery (¶, B cell)**  severe MZ B-cell differentiation block | High levels of IgM and IgE, slightly low levels of IgG1/2/3 and IgA (¶, B cell-extrinsic)  Impaired TI and normal to impaired TD Ag responses** | Mild decrease of thymic Tregs (§, mTEC),  Increase of splenic Tregs (¶, DC)  impaired thymic and peripheral iNKT cell development (§, mTEC) | **Severe reduction in cDC2 numbers in the spleen**** (mostly ¶), thymus (§) and BM | Myeloid hyperplasia,  splenomegaly (extramedullary hematopoiesis) (§) | [MGI:2179545](https://www.informatics.jax.org/allele/MGI:2179545)  [MGI:2179544](https://www.informatics.jax.org/allele/MGI:2179544) |
| *IKKA* | Ikka^-/-^ | AR complete IKK-α deficiency  (Cocoon syndrome) | ([*Chuk^tm1Ver^*](https://www.informatics.jax.org/allele/MGI:2386272)*)*  C57BL/6J  [Chuk^tm1Mka^](https://www.informatics.jax.org/allele/MGI:1857768)  C57BL6/J  [Chuk^tm1Aki^](https://www.informatics.jax.org/allele/MGI:1857735)  C57BL/6 | Strongly impaired: perinatal death (developmental defect) | **Epidermal keratinocyte differentiation defects:** Cocoon-like shape with shorter limbs, a fused tail, and a shiny skin without whiskers  **skeletal morphogenesis defects:** craniofacial, skeletal and tooth abnormalities | Severe infiltration: liver and pancreas (§, mTEC)  ANA and organ-specific auto-Abs (§, mTEC) | Abnormal medulla formation,  almost complete absence of UEA-1^+^ mTEC,  **very weak Aire expression** | No PP formation | Normal B-cell development in the BM  partial decrease in the number of peripheral mature B cells post transitional T_2_ stage | Partial decrease in basal IgG1/2/3, IgM, IgA levels  Impaired response to TD antigens | Reduced frequency and number of Tfh cells (§, mTEC);  Reduction of thymic DC (¶)  Reduction of thymic and peripheral Tregs | NA | - | [MGI:3714161](https://www.informatics.jax.org/allele/MGI:3714161)  [MGI:1857735](https://www.informatics.jax.org/allele/MGI:1857735)  [MGI:1857768](https://www.informatics.jax.org/allele/MGI:1857768)  [MGI:2386272](https://www.informatics.jax.org/allele/MGI:2386272) |
|  | Ikka^A/A^ | AR IKK-α deficiency (kinase-deficient) | [Chuk^tm2Mka^](https://www.informatics.jax.org/allele/MGI:3045616)  129/C57BL/6 | Viable, normal lifespan | Normal skin and skeletal differentiation,  defective mammary gland epithelial cell maturation,  normal molar and incisor tooth development | Mild: liver, lung and kidney | Disorganized thymic medulla,  very small numbers of UEA-1^+^ mTECs,  **very weak Aire expression** | **Impaired LN development, absence of PP** (§)  Absence of FDC network in the spleen (§) | Partial decrease in the number of mature IgD^+^ B cells in the spleen and LN | Impaired TD responses | Low Treg numbers | NA | - | [MGI:3045616](https://www.informatics.jax.org/allele/MGI:3045616) |
| *NIK* | Nik^-/-^ | AR NIK deficiency | Map3k14^tm1Rds^  C57BL/6 | Impaired (death between 12 and 24 weeks due to infections and wasting) | No | Severe T-cell and eosinophilic infiltration: liver, lungs, pancreas, salivary glands, spleen, and skin, anti-tissue auto-Abs (§, mTEC) | Disorganized thymic medulla,  very small numbers of mTECs,  **low Aire expression** | Partial reduction of LN, complete absence of PP  absent B cell follicles in LN, absent FDC networks in spleen  impaired MZ formation in spleen | Very small numbers of mature peripheral B cells (T2, follicular B cells, and MZ B cells) | Very low basal serum IgA levels (¶)  very weak TI (§) and TD responses | Reduced thymic, and peripheral Tregs  reduced frequencies and numbers of Tfh cells (§, mTEC) | Normal thymic and peripheral DC numbers  Reduced splenic cDC2 numbers | High numbers of monocytes and eosinophils | [MGI:2387668](https://www.informatics.jax.org/allele/MGI:2387668) |
|  | Nik^aly/aly^ |  | Map3k14^aly^ (G855R mutant)  C57BL/6 | Strongly impaired (death 15 days after birth) | No | **Severe T-cell infiltration**: pancreas, lungs, liver, salivary and lacrimal glands, stomach; no anti-tissue auto-Abs detected (§) | Disorganized thymic architecture, no medulla formation  very small numbers of UEA-1^+^ mTECs  **very weak Aire expression** | **Lack all LNs and PP**  disorganized splenic architecture, **absent lymphoid follicles, and FDC networks in spleen** (§)  **No MZ formation in the spleen** | Small numbers of mature B cells in BM, spleen, and peripheral blood, including MZ and follicular B cells (¶, B cell) | Low serum IgM and IgG levels (¶), no IgA (B cell-extrinsic)  impaired TD response (§) and TI response | Reduced thymic Tregs, low peripheral Tregs  impaired thymic and peripheral iNKT cell development | Normal thymic DC numbers | Impaired mammary gland development (§) | [MGI:1858522](https://www.informatics.jax.org/allele/MGI:1858522) |
| *TRAF3* | Traf3^-/-^ | Not reported | Traf3^tm1Bal^  C57BL6/J | Postnatal lethality: death10 days after birth (neonatal wasting) | No (<day 10) | Kidney and liver with anti-tissue auto-Abs; dsDNA autoantibodies (¶, B) | Normal thymic size and architecture  Normal numbers of AIRE-expressing mTECs | Spleen atrophy with normal architecture  *NB: splenomegaly and expanded B cell follicles in Traf3 B or T cell conditional KO* | Impaired transition from pro-B to pre-B stage in the BM  (¶, B cell)  *NB: large numbers of mature B cells (LN and spleen) with increases in the numbers of follicular, MZ, and transitional T2 cells (Traf3 B cell conditional KO)* | Impaired TD (¶, T cells) but normal TI responses  *NB: Hyper-IgA/M/G2/G3, normal TD and enhanced TI Ab responses (Traf3 B cell-conditional KO)* | Reduced thymocyte numbers with reduced double positive thymocyte proportions  *NB: Increased Tregs and TFh, and reduced iNKT cells (Traf3 T cell conditional KO)* | NA | Hypoglycemia, hypercortisolemia  depletion of peripheral leukocytes | [MGI:2135257](https://www.informatics.jax.org/allele/MGI:2135257) |
|  | Traf3^+/-^ | AD TRAF3 haploinsufficiency | Traf3^tm1Bal^  C57BL6/J | Viable, normal lifespan | No | NA | NA | Normal spleen size (B-cell conditional KO) | large number of mature and MZ B cells (B cell-conditional KO) | NA | NA | NA | NA | [MGI:2135257](https://www.informatics.jax.org/allele/MGI:2135257) |
| *BAFFR* | Baffr^-/-^ | AR BAFFR deficiency | Tnfrsf13c^Bcmd1^ or Bcmd1^A/WySnJ^ C57BL/6  Tnfrsf13c^tm1Mass^  C57BL/6 | Viable, normal lifespan | No | No | Normal | Normal LN development  Small GC in the spleen with absent MZ B cells, almost normal mature FDC network  normal splenic architecture with very few mature splenic and LN B cells | Normal BM B-cell development  very small numbers of mature B cells in the periphery, LN, and BM (including follicular and MZ B cells)  almost complete **B-cell developmental block from the T1 to T2 transitional stage** | Reduced basal IgG1/2/3, IgM, partial decrease in serum IgA levels  **impaired TD** **Ag responses** and normal TI Ag responses  **impaired survival of mature B cells *in vivo*** | Normal T-cell development | NA | - | [MGI:2389403](https://www.informatics.jax.org/allele/MGI:2389403)  [MGI:3054891](https://www.informatics.jax.org/allele/MGI:3054891) |
| *LTBR* | Ltbr^-/-^ | AR LTBR deficiency | Ltbr^tm1Kpf^ C57BL/6 | Viable, normal lifespan | No | Mild: lungs, liver, pancreas, salivary and adrenal glands, kidneys (CD4^+^ and B cells), and anti-tissue auto-Abs (§) | Disorganized thymic medulla  small numbers of UEA-1^+^ mTECs  Normal Aire expression** | **Complete absence of peripheral LN and PP** (§, FRC and LEC)  abnormal splenic architecture, aberrant GC formation without FDC networks, no MZ in spleen (§), **absent splenic FRCs (MRC, FDC and TRC)** (§, FRC) | Peripheral B-cell lymphocytosis with normal B-cell subtype proportions  Absence of the splenic MZ B-cell population | Low basal serum IgE levels  **Impaired Ab responses to TD Ags,** normal TI responses | Normal thymic T-cell development. Large numbers of peripheral T cells  Slightly low numbers of peripheral iNKT cells (§), normal in thymus | Normal thymic DC numbers  Low numbers of splenic cDC2 (¶, DC) | Splenomegaly | [MGI:2384140](https://www.informatics.jax.org/allele/MGI:2384140) |
| *CD40* | Cd40^-/-^ | AR CD40 deficiency (HIGM syndrome) | Cd40^tm1Kik^  C57BL/6 | Viable, normal lifespan | No | No | Normal thymic architecture  low to normal numbers of UEA-1^+^ mTECs**  Normal Aire expression | Normal lymphoid follicle structure in LN or spleen  No GC formation | Normal BM B-cell development  Normal numbers of peripheral B cells. **No memory B cells,** low MZ B-cell count | **Normal IgM** and IgG3 levels, low IgA , IgG1/2 levels and no IgE in serum  **impaired Ab response to TD antigens**, normal TI responses | Normal T-cell development  Low levels of Tregs in blood, thymus, spleen and LN (¶, T cells) | Normal DC developmentwith impaired maturation | - | [MGI:1857457](https://www.informatics.jax.org/allele/MGI:1857457) |
| *RANK* | Rank^-/-^ | AR RANK deficiency | Tnfrsf11a^tm1Imx^  C57BL/6 | Neonatal death between 3 and 5 weeks of age (inability to nurse) | No | Mild: lung and liver (stroma-dependent), anti-tissue auto-Abs (stroma-dependent) | Normal thymic architecture,  very small numbers of UEA-1+ mTECs  Very weak Aire expression | No lymph nodes (§), almost **normal PP development** (smaller numbers and size), and general splenic architecture (normal MZ and FDC network) | Impaired early B-cell development in the bone marrow (pro-B to pre–B-cell stages) (¶)  Partial decrease in the number of mature B cells in the spleen and periphery (B cell-extrinsic) | Impaired TD response to Ag (B cell-extrinsic) | Normal thymocyte development | Normal DC numbers in spleen, skin, and thymus | **Severe osteopetrosis** (abolished osteoclast development),  defective lactating mammary-gland maturation,  splenomegaly, extramedullary hematopoiesis | [MGI:1860238](https://www.informatics.jax.org/allele/MGI:1860238) |

Abs, antibodies; Ag, antigen; ANA, antinuclear Abs; auto-Abs, autoantibodies; BM, bone marrow; DC, dendritic cells; FDC, follicular dendritic cells; FRC, fibroblastic reticular cells; GC, germinal centers; KO, knockout; LEC, lymphatic endothelial cells; LN, lymph nodes; MRC, marginal reticular cells; MZ, marginal zone; mTEC, medullary thymic epithelial cells; PP, Peyer’s patches; TD, T-dependent; TI, T-independent; TRC, T-zone reticular cells; Tregs, regulatory T cells.

¶ Hematopoietic cell-intrinsic;

§ Stromal cell-intrinsic

* No information available for heterozygous mice

** Heterogeneity between studies and/or genetic backgrounds

# References can be found at https://www.informatics.jax.org/allele/*MGI:number*
