# Supplementary material for: Human inborn errors of the alternative NF-κB pathway
Source: J Hum Immun. 2025 Nov 21;2(1):e20250104. doi: 10.70962/jhi.20250104 (PMC12829755; doi:10.70962/jhi.20250104)
Supplement: Table S3 — shows NFKB2 variant nomenclature and functional consequences. [file jhi_20250104_tables3.docx]

**Supplementary Table III: *NFKB2* variant nomenclature and functional consequences**

|  | Phenotypes and readouts by overexpression studies (HEK293T cells) | | | | Functional consequences in patients’ heterozygous cells (fibroblasts or MDDCs) | | |  |
| --- | --- | --- | --- | --- | --- | --- | --- | --- |
| Disease forms (based on biochemical data) | p100 basal expression | p100 activation | p52/p52 transcriptional activity  (“*p52 function*”) | IκBδ regulatory function  (“*IκBδ function*”) | WT/mutant p100 basal expression and localization | WT/mutant p100 activation | IκBδ regulatory function | Disease forms (based on patients’ cell or inferred) |
|  | Western Blot, confocal microscopy | p100 phosphorylation, processing to p52 (Western Blot using p100 plasmid) | RelA-mediated transcriptional repression (κB reporter assay using p100 plasmid) | p52/RelB cytoplasmic retention by WT/mutant p100 (confocal microscopy or κB reporter assay using p100 or C-ter plasmid) | Western Blot | WT/mutant p100 phosphorylation, processing to p52 (Western Blot) | WT p52/RelB cytoplasmic retention (confocal microscopy) |  |
| WT-NF-κB2 | + (cyto) | N | N | N | N/N (cyto) | N/N | N | WT |
| p52^LOF^/IκBδ^LOF^ | + (nucl) | - | - | - | N/N (cyto) or N/- | N/- | almost normal | p100/p52 haploinsufficiency |
| p52^GOF^/IκBδ^LOF^ | + (nucl) | - | +++ | - | NT/NT | NT/NT | NT | AD p52-GOF |
| p52^LOF^/IκBδ^GOF^ | + (cyto) | - | - | +++ | N/+++ (cytoplasmic accumulation) | impaired/- | +++ | AD IκBδ-GOF / DAVID syndrome |

“-“ means loss-of-expression or loss-of-function; “+++”, gain-of-function; cyto, cytoplasmic; MDDC, monocyte-derived dendritic cells; N, normal; NT, non-tested; nucl, nuclear.

All data are from Le Voyer *et al.,* Nature, 2023.
